# Leptin: A Correlated Peptide to Papillary Thyroid Carcinoma?

**DOI:** 10.4061/2011/832163

**Published:** 2011-10-05

**Authors:** Mehdi Hedayati, Parichehr Yaghmaei, Zahra Pooyamanesh, Marjan Zarif Yeganeh, Laleh Hoghooghi Rad

**Affiliations:** ^1^Obesity Research Center, Research Institute for Endocrine Sciences, Shahid Beheshti University of Medical Sciences, 1985717413 Tehran, Iran; ^2^Department of Biology, Faculty of Basic Sciences, Science Research Campus of Islamic Azad University, 1939614484 Tehran, Iran

## Abstract

*Introduction*. Leptin as an adipose-tissue-related peptide hormone contributes to the control of food intake, energy expenditure, and other activities such as cell proliferation. Therefore, association of leptin level with thyroid cancer has been suggested recently. Considering that thyroid cancer is the most common endocrine cancer, the aim of this study was evaluation of leptin levels in thyroid cancer. *Materials and Methods*. 83 patients with papillary thyroid cancer (35 males and 48 females) with 90 healthy persons as control group (40 male and 50 females) were selected. serum thyroxine, thyrotropin, and leptin levels were determined in both groups. As a body fat tissue affects leptin level, so height and weight were measured and body mass index was calculated too. *Results*. There was no statistically significant difference in age, serum Thyroxine, and Thyrotropin levels. BMI in women was more than in men in both groups. Serum leptin levels in thyroid cancer group were significantly higher than control group (*P* < 0.05). *Conclusion*. The results of this study showed an acceptable association between the hormone Leptin levels with papillary thyroid cancer, so it may be considerad as a correlated peptide which may help in the diagnosis or confirmation of thyroid cancer beside in other specific tumor markers.

## 1. Introduction

leptin with 16 kDa molecular weight mainly produces by white adipose tissue cells [1]. So its level is proportional to the adipose tissue mass [2]. leptin as a neuroendocrine hormone has effects on the glucose metabolism, sexual maturation, reproduction, pituitary-adrenal axis, immune system, thyroid, and growth hormones level [3–6]. The association between this neuroendocrine hormone with obesity and some cancers has been proposed. Probably this hormone is an important risk factor in carcinogenesis, because obesity itself can promote tumorgenesis and is a risk factor for cancer over time [7, 8]. On the other hand, leptin plays an important role in the oxidation reactions such as fatty acid oxidation [9] and angiogenesis [10]. There are many reports concerning the effect of leptin on stimulation of cell mitosis and its involvement in carcinogenic stages of breast, clone, prostate, lung, kidney, and ovary cells [11–16]. Studies have shown that leptin by increase of cell proliferation and inhibition of apoptosis is involved in creating certain types of tumors [17–19].

leptin acts through its receptor on the cell surface and its receptor expression also increases following the activity of PI3K/AKT pathway and increases the activity of antiapoptosis molecules such as Bcl-XL and XIAP [20]. In some cancer cells, expression of leptin receptor levels and stimulation by leptin will lead to increase of cell proliferation [21]. leptin stimulates expression of some molecules such as CyclinD1, CDK2 and c-Myc that result in cell cycle progression and cell proliferation [21, 22].

The Important molecular pathways, such as JAK/STAT3, PI3K/AKT, ERK/MAPK, in many cancer cells can be activated by leptin/leptin receptors [21–24]. Furthermore, leptin with the induction of VEGF and VEGF-R2 molecules expression plays an important role in the tumorigenesis [25]. These molecules are involved in many malignancies such as colon, stomach, endometrial, ovarian, and breast cancer [26–30]. Additionally, increased serum levels of leptin and its receptor have been associated with distant metastases, disease recurrence, and lower survival in patients with breast cancer [31]. Increased expression of leptin and its receptor in papillary thyroid cancer has been proved. This hormone probably through its receptor and activation of the PI3K/AKT pathway plays an important role in papillary thyroid cancer pathogenesis. It also seems that the oncogenic effects of leptin on papillary thyroid carcinoma cells are related to the stimulating cell proliferation and apoptosis inhibition. Involvement of thyroid hormones on basal metabolism and regulating appetite and weight control in many scientific reports is given explicitly [10, 32–34]. The most common endocrine malignancy is thyroid cancer, and papillary form of thyroid cancer is the most common type of thyroid cancer (80–90%) [32]. The aim of this study was determining the serum leptin levels in patients with papillary thyroid cancer and its comparison with healthy subjects.

## 2. Materials and Methods

### 2.1. Subjects

**Patients**

The case population consisted of 83 individuals, including 35 males and 48 females, 14 to 62 years (mean age 38.6 years) with papillary thyroid cancer (PTC). They were referred to Research Institute for Endocrine Sciences, Shahid Beheshti University of Medical Science. Also, 90 persons were selected as control group (40 male and 50 females) from referred to the laboratory with normal thyroid function tests (TSH: 0.3 = 3.5 mIU/L, T4: 4.5 = 12.5 *μ*g/dL, T = Up: 25 = 35% and T3: 75 = 210 ng/mL) with age, sex, BMI matched with case group. Both groups were also matched for age and sex. The participants were included in the survey after obtaining an informed consent. Also, the clinical examination was performed by endocrinologist. The diagnosis of PTC was confirmed by histopathologic documents. This study has been approved by Institutional Review Board and Ethics Committee of Obesity Research Center, Research Institute for Endocrine Sciences, Shahid Beheshti University of Medical Sciences.

### 2.2. Methods

Blood sampling was performed in both studied groups. For preparation of serum, 3 mL of whole blood was collected from antecubital vein in sitting position and was incubated 10 min in RT for coagulation. Then sera separated by 10 min centrifugation at 3000 rpm and the obtained sera were aliquoted in three 0.5 mL microtubes. The isolated serum samples from each individual were stored in 1 mL Eppendorf microtubes at −80°C (Japan's Sanyo C Company).

Anthropometric characteristics, including height and weight of patients and control group, were measured by height measuring scaled balance (Seca, German company); height with 0.5 cm and weight with 250 g sensitivity were reported. These data were used to calculate the body mass index (kg/m^2^). Demographic profiles, including age, and sex were also recorded. Those individuals, who were using drugs affecting thyroid function and obesity drugs, were excluded.

In groups thyroxine, thyrotropin, and leptin hormones were measured by ELISA method. The used kits were prepared from the Canadian company (DBC Company, Ontario, Canada). The ELISA reader was from Tecan Austrian Company and Sunrise Model. Human thyrotropin and leptin hormones were determined based on sandwich ELISA method, whereas a thyroxin hormone was measured according to the competitive EIA method. The sensitivity of thyroxine, thyrotropin, and leptin kits was 0.6 *μ*g/dL, 0.1 mU/L, and 0.4 ng/mL, respectively. Additionally, the coefficients of variation for these assays were 6.2%, 7.1%, and 6.5%, respectively.

#### 2.2.1. Statistical Analysis

According to the normal distribution of data obtained by testing Kolmogorov-Smirnov (KS) (*P* = 0.68 for case group and *P* = 0.52 for control group), the frequency, mean and standard deviation were used to describe characteristics. All the data were in normal distribution (except leptin after normalization). The independent *t*-test was used to compare mean (except leptin with geometric mean and CI 95%) of variables between two groups. Comparison of qualitative data was done with Chi-square test. Further, data was analyzed using statistical software (SPSS 15), and significant level was considered at 0.05.

## 3. Results

Demographic profiles and anthropometric characteristics of participants are provided in Table 1. The results of thyroid hormones test, including thyroxine and thyrotropin in both control and patients groups, are given in Table 2. Since the leptin hormone secreted from adipose tissue is different in male and female, therefore the different levels of measured leptin hormone in two groups are shown in Table 3 (gender based). Height, weight, and body mass index between males and females of both groups were significant (*P* < 0.05). In addition, a significant difference (*P* < 0.05) was observed between the leptin hormone levels in males and females in both healthy and cancer groups. The amount of leptin hormone in cancer patients was higher than that in normal individuals, significantly (*P* < 0.05).

## 4. Discussion

Our data showed that the serum leptin levels of Iranian patients with papillary thyroid carcinoma were significantly higher than those in control group subjects. This increased level was observed in both males and females with papillary thyroid carcinoma. As this increased level was observed in both gender and different ages, so it could be related to thyroid carcinoma and it is independent of sex and age. Even though in this study the leptin level was higher in females than males in both groups, this is probably related to more adipose tissue mass in women. Both leptin and thyroid hormones cause thermogenesis and reduce body weight therefore maybe it is considered as a first association between the two hormones. The hunger reduces the leptin and thyroid hormones levels [35]. High levels of thyroid hormones decrease leptin expression in adipose tissue. But the most studies have not shown significant changes in leptin levels in hypothyroidism and hyperthyroidism disorders [36, 37]. However, increased leptin level in postpartum thyroiditis has been reported [38]. Akinci et al. reported that leptin levels increased in papillary thyroid carcinoma in Turkish population [39]. But in their study only 34 cases were investigated, the status of thyroid function in patients and healthy group was not evaluated, and age-matching was not considered [39]. In our study, 83 persons were matched for age, sex, and BMI. Assessing thyroid function in patients and healthy individuals was performed, and no significant difference was observed in both groups.

In both above studies, BMI in women was higher than in men, which was quite predictable. In both studies leptin levels in women were higher than those in men that is because of increased fat mass in women. In one study Cheng et al. showed that expression of leptin and/or leptin receptor in papillary thyroid cancer was associated with neoplasm aggressiveness, including tumor size and lymph node metastasis [40]. Interestingly, in another study, Uddin et al. demonstrated that leptin plays an important role in papillary thyroid cancer pathogenesis through PI3K/AKT pathway via its receptor (Ob-R) and is a potential prognostic marker associated with an aggressive phenotype and poor survival [32].

One of the limitations of our study was inability to followup the patients after surgery. Therefore, reduction or normalization of high leptin levels in thyroid cancer patients was not assessed. However, a significant increase of serum leptin levels in Iranian patients with papillary thyroid carcinoma maybe used as a reliable marker to diagnose or confirm papillary thyroid cancer. In addition if the leptin levels in cancer patients decrease after thyroidectomy, it will be used for the followup treatment, possibly. So a before-after study is recommended for future investigations instead of case control study. Thus, leptin level measurement can be used to followup the treatment of patients.

Strongly high leptin level in papillary thyroid cancer patients in comparison with health subject potentially suggests leptin as a peptide marker of papillary thyroid cancer. It means that adipose tissue secreted hormones, proteins, and peptides potentially may have application in diagnosis, confirmation, and/or treatment followup.

## Figures and Tables

**Table 1:** Anthropometric characteristics of participants.

Group	Sex	Height (cm)	Weight (kg)	BMI (kg/m^2^)	Mean age (year)
Control	Female (50)	159.5 ± 12.1	61.9 ± 0.3	24.5 ± 2.3	38.1 ± 12.5
	Male (40)	169.2 ± 10.5	66.3 ± 0.2	23.2 ± 2.0	37.9 ± 15.8
Case	Female (48)	160.2 ± 11.6	63.7 ± 0.3	24.9 ± 2.7	39.1 ± 13.7
	Male (35)	170.4 ± 11.3	68.4 ± 0.2	23.7 ± 2.4	37.5 ± 17.0

**Table 2:** Serum levels of thyroxin and thyrotropin hormones in participants.

Group	Thyroxin (*μ*g/dL)	Thyrotropin (mU/L)
Control	9.1 ± 2.9	2.4 ± 1.2
Case	8.9 ± 3.0	2.6 ± 1.0

**Table 3:** Serum leptin levels in participants.

Group	Females serum leptin level (ng/mL)	Males serum leptin level (ng/mL)
Control	4.3 ± 6.9	2.2 ± 5.6
Case	19.6 ± 23.3 (*P* < 0.05)	10.4 ± 17.3 (*P* < 0.05)
